# The origins of the domesticate brown rat (*Rattus norvegicus*) and its pathways to domestication

**DOI:** 10.1093/af/vfab020

**Published:** 2021-06-19

**Authors:** Ardern Hulme-Beaman, David Orton, Thomas Cucchi

**Affiliations:** 1 Department of Archaeology, Classics and Egyptology, University of Liverpool, 12–14 Abercromby Square, Liverpool, L69 7WZ, UK; 2 BioArCh, Department of Archaeology, University of York, York YO10 5DD, UK; 3 Archaeozoology, Archaeobotany, Societies, Practices, Environments (AASPE-UMR7209), CNRS, National Museum of Natural History (MNHN), Paris, France

**Implications**

- Historical evidence indicates that brown rats went through a series of human-influenced and/or controlled-breeding events at different times and locations: Japan in the 1600 to 1700s, Europe in the early 1800s, and North America in the mid-1800 to early 1900s.

- The European and Japanese controlled-breeding events may be considered domestication events, whereas the later events from the mid-1800s onward might be considered selective breeding of an already domesticated animal.
- Each event appears to have been for a different purpose: Japanese rats were pets and ornamental; early European breeding was in the first instance for blood sports and food sources for captive carnivores; North American selective breeding was for laboratory use.
- Modern examination of domestic brown rats has almost exclusively focused on laboratory strains, which stem from a limited source and there has been little to no exploration of pet or fancy rat populations.

## Introduction

**Figure 1.**
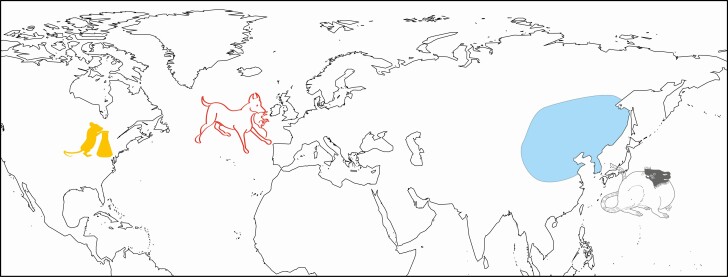
Map indicating the assumed native distribution of *Rattus norvegicus* (in blue) and locations of major rat domestication processes (marked with depictions). From East to West: Hooded rat depiction adapted from an image within the 1700s Japanese rodent breeding guide book, the *Chinganso-date-gusa* (1787); Rat-baiting dog depiction adapted from Mayhew (1851)*London labor and the London poor* illustrating blood sport activities from which European domestic rats arose; laboratory rat silhouette representing the postdomestication selective breeding of rats for laboratory inbred strain development at the Wistar Institute of Philadelphia in the early 1900s.

The brown rat (*Rattus norvegicus*) is one of the most pervasive and familiar species across the globe. Its familiarity in society and experimental lab work belies a complex history (Figure 1), which is exemplified by its contradictory name; it is not always brown as additional color morphs regularly occur in some populations (Aplin et al., 2003), and there is no fossil evidence for any *Rattus* species from Norway, from which its binomial “*norvegicus*” derives (Berkenhout, 1769; Lindsey and Baker, 2019). Biologically, it is extremely well understood as it forms one of the major mammalian model lab species and is only rivaled by the mouse (*Mus musculus*) (Hedrich, 2019). However, extraordinarily little is known about the brown rat’s native origins and natural/wild behaviors (Hulin and Quinn, 2006; Ness et al., 2012; Zeng et al., 2018). The vast majority of research conducted on this species is focused on laboratory work and observations of behaviors in domestic and laboratory populations; wild studies are almost exclusively focused on invasive commensal populations and those occupying human environments where ecological pressures and competitive interactions with other species differ from its likely native range (e.g., ecological and evolutionary studies such as Figgs, 2011; Kajdacsi et al., 2013; Puckett and Munshi-South, 2019). As such the dispersal history, evolution and origins of the brown rat in the lead up to and eventual domestication are all in considerable need of investigation. Here we provide a brief review of this biologically important and ecologically influential species and examine the processes by which it was domesticated.

## Brown Rat Origins

The origins of the brown rat are far from clear with its earliest association with humans obscured by a lack of direct evidence. Fossil evidence for the brown rat is scarce, as it is for many species of the *Rattus* genus, and identification of fossil specimens to species level can be extremely challenging (Pagès et al., 2010; Hulme-Beaman et al., 2019). The divergences of *R. norvegicus*, its natural ecology and indigenous precommensal range are subject to much uncertainty as a result (Hedrich, 2019). Genetic evidence suggests that the brown rat diverged from the other major Eurasian *Rattus* species between 0.9 and 2.9 million yr ago (Teng et al., 2017; Zeng et al., 2018), sometime between the early and middle Pleistocene. Genome-wide studies of the brown rat and its sibling species the Himalayan field rat (*Rattus nitidus*) indicate these two species diverged sometime in the Middle Pleistocene following large-scale climate fluctuations, but that early divergence was followed by extensive and multiple introgression events (Teng et al., 2017). Middle to late Pleistocene evidence for *Rattus* spp. extends across Eurasia, with a *Rattus* species (*Rattus cf. haasi*) present in the eastern Mediterranean up until at least the Last Glacial Maximum (**LGM**) (e.g., in the Zuttiyeh cave, Israel: Tchernov, 1968; Yarimburgaz Cave, Turkey: Santel et al., 1998; Qesem Cave, Israel: Maul et al., 2016). However, both the dating and the taxonomic relationship between the modern brown rat species and these Pleistocene rats remains unclear (Tchernov 1968). Some fossil evidence also identifies the presence of the brown rat (stricto sensu *Rattus norvegicus*) in southern China during the middle and late Pleistocene (Wu and Wang, 2012), but it is unclear how these fossil specimens compare with closely related species such as *R. nitidus*, which is often described as having a more southerly range (Aplin et al., 2003).

The indigenous distribution of the brown rat is often cited as the northern regions of China, Mongolia, and/or south-eastern Siberia (Figure 1), and its ability to survive well in temperate climates is used to support this (Hedrich, 2006; Puckett et al., 2016). Although not explicitly stated, reference to indigenous range in most modern studies probably refers to its natural range after the LGM, as climate and environment in the northern region of China has changed considerably over the late Pleistocene and Quaternary (Yang et al., 2004). Discussions of the brown rat’s temperate climate adaptations and native range, however, do not appear to consider paleoclimates directly. There is a large gap in the record and knowledge surrounding the period and process during which the brown rat adapted to human environments in Chinese prehistory (see below). From its native distribution in East Asia, the brown rat was later transported globally, most likely on ships (Puckett et al., 2020). This appears to have occurred sometime in the 1700s based on European and North American documentary accounts (Hedrich, 2019) and has led to a global distribution with multiple invasive and commensal populations. In large part, efforts to model brown rat native distributions are hampered by an inability to robustly assess which commensal and translocated populations are surviving in extreme climates because of human resource exploitation and which populations could survive such climates in the absence of humans.

A number of different genetic studies conducted on modern specimens have suggested varying commensal and precommensal origins within East Asia, including both Southeast and/or Northeast Asia (Song et al., 2014; Puckett et al., 2016; Zeng et al., 2018; Puckett and Munshi-South, 2019), but those studies do not include any ancient specimens. As such, the native range of the brown rat remains debated, but generally restricted to eastern Asia. Genomic analyses have also been used to identify probable demographic expansions that might be associated with advances into human-commensal niches and further translocation events (Zeng et al., 2018; Puckett and Munshi-South, 2019). However, the results vary widely with some suggestions of expansions dating to ~800 BP (Puckett and Munshi-South, 2019) and others of expansions dating much earlier to between 3000 and 1800 BP (Zeng et al., 2018). These early dates of expansion do not at all match historical accounts of new arrivals of rats to Europe in the 1700s, which describe in some detail a new species of larger rat aggressively competing with and extirpating existing rat populations in France, England, Ireland, and Denmark (Buffon, 1760; Pennant, 1768; Rutty, 1772; Smith, 1772; Winge, 1908 referring to notes from 1755 by Urne). Without ancient specimens, these genetic studies remain limited and robust species divergence dates and assessment of native ranges, pre- and post-LGM, will remain poor until evidence is bolstered by extensive and combined zooarcheological and ancient DNA analyses (e.g., as has been done with mice; Cucchi et al., 2020).

## Earliest Archeological Evidence

Archeological evidence for the brown rat is extremely poorly documented (Armitage, 1994; Ervynck, 2002). This is largely due to the brown rat’s innate fossorial (burrowing) behavior, which leads to it being a major contaminant in archeological contexts and a general source of taphonomic disturbance (Armitage, 1994). Added to this are the difficulties of identification with regards to other commensal rats: although intact crania of black and brown rats are readily distinguishable, metrical indices on the mandible (Armitage et al., 1984) show overlap between species (Walker et al., 2019), and postcranial morphological criteria (Wolff et al., 1980; Ervynck, 1989) are relatively unreliable (Koski, 2019). Although black rats are typically significantly smaller than brown rats when the two are found in the same environment, the former may be larger—and may exploit a wider niche—when the latter is absent (Ervynck, 1989; Armitage, 1994). The resulting variability and potential overlap renders size an unreliable criterion for distinguishing archeological specimens. The identification of fossil specimens of *Rattus* species is particularly difficult, especially in regions where multiple *Rattus* species are sympatric (Hulme-Beaman et al., 2019); this is well demonstrated by the regular occurrence of misidentification of *Rattus* species in modern field caught studies and museum collections (Pagès et al., 2010).

The earliest record of brown rats in archeological contexts confirmed with advanced morphological analyses (geometric morphometrics) derive from central or northern China dating to the early Neolithic and the development of agriculture (7000 to 9000 BP—A. Hulme-Beaman et al., in preparation). These were found in direct association with humans and human refuse middens, which indicates a likely commensal relationship for this population. The next archeological evidence for commensal brown rats, secure both in dating and taxonomic identification, is distant in time and space from the species’ native range, deriving from an 18th-century shipwreck off the coast of Corsica (Vigne and Villié, 1995). Earlier reported European finds, such as at 14th-century Tarquinia, Italy (Clark et al., 1989), require direct dating and confirmation of taxonomic identity. For now, the early direct evidence for the emergence of commensal behaviors in brown rat populations is extremely limited, and the process of adaptation to human environments is poorly understood. There is no direct archeological evidence for the domestication of brown rats or their maintenance in captivity; historical accounts of rat keeping and breeding are therefore the best evidence for itsdomestication.

## The Domestication of the Brown Rat

The pathway to domestication (see Sidebar 1) for the brown rat might not be so clear as it first seems given selective breeding of rats for laboratory use and its intense commensal relationship with humans. In addition to breeding in close association with humans in commensal populations, the brown rat was managed and deliberately bred in controlled circumstances or direct captivity under at least three different conditions and similarly for three different purposes: rat-baiting rats (Mayhew, 1851), fancy rats (Kuramoto, 2011), and biomedical laboratory rats (Richter, 1954) (Figure 1). Each different purpose could align somewhat with a different proposed pathway to domestication as the selective pressures would be different under each circumstance (Zeder, 2012). An important factor in this is also the possibility of different pathways to domestication occurring for different populations of rats in space and time. This is most notable in the domestication of brown rats whereby major laboratory strains come from different populations bred into inbred laboratory strains at different times.

**Sidebar 1. Pathways to domestication**

Within studies of the process of domestication, three major pathways have been proposed (Zeder, 2012): commensal pathway; prey pathway; and directed pathway. Each of these pathways has a different starting point and likely initiated by different agents in the process. The commensal pathway to domestication is initiated by the nonhuman agent in the process and occurs when other animals are attracted to human environments and undergo a prolonged period of habituation with humans; an example of this might be the cat whereby the progenitors of modern domestic cats might have been attracted to human environments due to human resources or the other commensal species that consume them. Other possible examples of the commensal pathway to domestication include the pig and the dog (Zeder, 2012). The prey pathway to domestication occurs with initiation by humans and is primarily focused on large prey species and occurs when humans increasingly manage wild game by encouraging their proliferation and then changing their demographics; this culminated eventually in determining which animals breed through herd management. It is thought to have likely occurred in the domestication of sheep (*Ovis aries*), goats (*Capra hircus*) and cattle (*Bos taurus*). Finally, the directed pathway to domestication is primarily instigated and managed by humans and involves the specific selection of individuals for breeding; once humans have an idea and familiarity with other domestic animals then directed pathway domestication can occur rapidly, with deliberate purpose. Animals that might have followed the directed pathway to domestication are recently domesticated species such as foxes (*Vulpes vulpes*), mink (*Mustela vison*) and chinchilla (*Chinchilla lanigera*). These pathways are not strict and aspects of each may cross over, but each may come with its own underlying selective pressures, which might then influence the course of their domestication.

The earliest evidence for a form of rat domestication comes from Japan where a tradition of keeping fancy rats emerged during the Edo period (1603 to 1868) (Serikawa, 2004; Hedrich, 2006). It is likely that these fancy rats derived from the brown rat (Serikawa, 2004; Hedrich, 2006; Kuramoto, 2011), though other species cannot be fully discounted (e.g., the Asian house rat [*Rattus tanezumi*] or another rat within the *Rattus rattus* species complex) because modern remnants of these Japanese fancy rats have not been identified (Kuramoto, 2011). It is also not clear as to whether Japan is part of the species’ native range or whether they arrived as commensal animals and if so, when this was. However, the early breeding of rats in Japan is very clearly demonstrated by two early breeding guides dating to the late 1700s (the *Yoso-tama-no-kakehashi* of 1775 and the *Chinganso-date-gusa* of 1787, Figure 2A), and many of the color morphs and patterns described are found in domestic brown rats today (Kuramoto, 2011). The early documentation of Japanese rodent breeding indicates that such breeding had been carried out as early as 1654 (Serikawa, 2004; Hedrich, 2019). There is also some indication that similar fancy rat breeding took place in China around the same time, or even possibly earlier, with old Chinese stories about fancy rodent breeding referred to in *Yoso-tama-no-kakehashi* (Kuramoto, 2011), but the direct relationship of these stories to specific breeding or domestication and the early occurrence of this needs further exploration. Furthermore, there are descriptions in the *Chinganso-date-gusa* of fancy mice being traded to Japan (Yonekawa et al., 1982) and as a result, the possibility of earlier fancy rat breeding in China cannot be excluded. This occurrence of rat domestication with specific selection for certain desirable traits might be considered to fall under the “directed pathway” to domestication, whereby people could have used their prior knowledge of management of domestic animals to “fast-track” the domestication process (Zeder, 2012).

**Figure 2.**
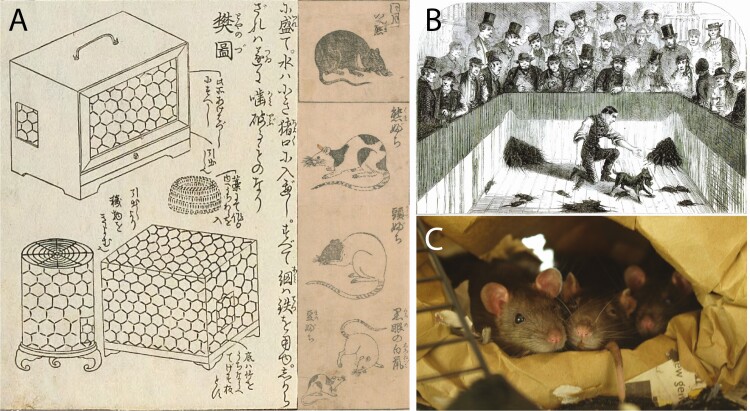
Panel of domestic rats. (A) Composite image of rat and mouse keeping cages from the *Yoso-tama-no-kakehashi* (1775) and four images of different color morphs of rats from the *Chinganso-date-gusa* (1787). (B) Rat-baiting event depicted from Mayhew (1851)*London labor and the London poor*. (C) Three female fancy rats displaying gregarious social behavior (photo credit Robert Lachlan).

Given Japanese isolation under the policy of *sakoku* from the 1630s to 1853, these fancy rats are unlikely to have contributed to rat populations elsewhere before the late 19th C, if at all—although commensal rats from Japan may have spread to the Aleutians via a shipwreck as early as 1780, according to a Russian account from 1826/1827 (Khlebnikov, 1979). Trained rats were displayed in Paris in 1667 according to the revised German edition of Gesner’s *Historia Animalium* (Gesner and Horst, 1669), but this anecdote appears in the entry on *mure domestico majore*, conventionally taken to refer to the black rat. Writing in Paris almost a century later, Buffon (1760) noted that it was “only nine or ten years” since the brown rat had appeared in the environs of the city.

Rats have also been kept as food and sport animals, though not for human consumption, and it appears that the process of managing such stocks may have given rise to most modern domestic rats (Lindsey and Baker, 2019). Within England, France, and later North America, brown rats were regularly bred from the early 1800s for sport with dogs in rat-baiting events (Richter, 1954); a single dog could kill up to 100 rats in a single timed round (Figure 2B, the number often decided by the dogs weight; Drabble, 1948) and, as such, large numbers were required (Mayhew, 1851). Albino individuals were removed from this breeding process and kept separately for show and further selective breeding (Mayhew, 1851), and Richter (1954) suggests that many modern domestic rats derived from this stock.

Some further accounts from rat catchers and fancy rat breeders in the London area in the 1800s suggest albino rats were wild caught, with one rat catcher, Jack Black, describing catching his first white rat wild in Hampstead, UK, and catching black color morphs in Regent Street, London, before breeding directly from them (Mayhew, 1851). Notably, Jack Black reports selling his tame and fancy rats widely and even internationally, with some 300 being sold to buyers in France (Mayhew, 1851).

Amongst the earliest captive rat closed colonies (i.e., those that are self-sustaining and not replenished with new animals) are those recorded from 1856 in the Jardin des Plantes, Paris (Lindsey and Baker, 2019). This colony was noted as consisting of hooded brown rats (white with a black head), was set up to feed the reptiles housed in the gardens, and was maintained until 1988 (Hedrich, 2019). Its foundation date closely matches that of the rat-baiting events and early selective breeding of the 1800s (Mayhew, 1851), so could follow Richter’s (1954) interpretation that modern stocks derive from such activities. As the colony consisted of individuals with a nonwild color morph, this population had probably undergone a number of selective breeding events already, reflecting the activities of local fancy rat breeders (Mayhew, 1851). This colony was not selectively bred for laboratory use though until much later and it was only in the 1980s that rats from it were taken and developed into an inbred laboratory strain for experimentation (Hedrich, 2019). Having been initiated via direct capture by humans, this process has obvious parallels to the Japanese fancy rats and the “directed pathway” to domestication. Yet in terms of population management for rapid consumption, and the indirect selective pressures likely to have been experienced by these managed populations, this domestication process arguably has some commonalities with the “prey pathway” (cf. Zeder, 2012).

Brown rats were among the earliest mammal species used specifically for laboratory experiments (Richter, 1959). The earliest use of the brown rat as a laboratory animal appears to have emerged in the early 1800s with the use of albino brown rats in dietary studies (Savory, 1863) and studies of the adrenal glands (Philippeaux, 1856). From that point onward, the brown rat was used in a range of different studies including specific breeding experiments in the late 1800s (Crampe, 1877, 1885), presumably with animals originating from rat-baiting activities. By the early 1900s, Henry H. Donaldson at the Wistar Institute of Philadelphia began the first breeding programs to establish specific laboratory inbred rat strains (Hedrich, 2019). Therefore, although there were closed colonies prior to 1900 such as that of the Jardin de Plantes, they were not bred with intent for laboratory purposes and it is only with the rat strain developed by Henry H. Donaldson that the first laboratory rats emerged. Even though the brown rat is often cited as being domesticated as a laboratory animal (e.g., Richter, 1959; Gibbs et al., 2004), from the perspective of wider domestication studies it might be considered that the specific breeding of rats for laboratories was largely secondary to the initial domestication process, which appears to have commenced with stock management and breeding of fancy rats (e.g., Mayhew, 1851). Breeding rats for laboratory use therefore is more comparable with the breeding of dogs for specific roles in human society and probably started with a rat population that had already been selectively bred and in some cases was maybe even docile.

## Origins of Modern Domestic Lineages

A number of genetic studies have been carried out on the different lineages of domestic rats to understand their relatedness (Canzian, 1997; Thomas et al., 2003; Puckett et al., 2018). Those genetic studies found that there is a large amount of genetic diversity amongst the domestic strains (Canzian, 1997; Thomas et al., 2003), though notably not nearly as much as in domestic mouse populations (Ness et al., 2012). In particular, these studies found that the strains from the likely oldest closed colony in the Jardin de Plantes are the most divergent (Canzian, 1997). This would further suggest multiple domestication events or early separation of breeding lineages. More recent work examining nuclear genomes at higher resolutions suggests the diversity found within combined inbred strains is moderate relative to the diversity within wild populations (Puckett et al., 2018). This study also found all laboratory strains examined (25 of >500) derived from a single ancestral source from a likely small and unknown geographic region (Puckett et al., 2018). Furthermore, the different strains show little evidence for clustering, which suggests that there were not multiple domestication events, but this might be due to extensive admixture amongst the progenitors of the different strains with the Wistar strain (Puckett et al., 2018). However, the rats being examined in these studies all derive from laboratory inbred strains, and it is unclear as to whether pet strains have been examined. The development of pet fancy rat breeds is unclear and rarely discussed in these papers. Although it might easily be assumed that pet strains derive from early laboratory breeding prior to the development of inbred strains, in fact the reverse is likely true and pet rats may harbor unknown diversity. The ancient Japanese strains of domestic rat have not been identified, nor have they been systematically looked for. This would suggest further research is required that incorporates rats bred for pet keeping and not just laboratory use.

## Effects of Human-Associated Adaptation and Domestication

The nature of brown rat domestication presents an interesting case for tracking both the unintentional and intentional effects of domestication, but also likely human-induced adaptations that may have occurred prior to domestication. The effects of domestication on any one species, let alone one specifically bred for experimental use, are extremely wide ranging and cannot all be addressed here (for further review of differences between laboratory and wild rats, see Modlinska and Pisula, 2020), but some major elements will be outlined and described which are relevant to the concept of domestication more generally.

A number of studies identify differences in commensal brown rat populations that indicate that the animals from which domestic lineages derive had already undergone some degree of adaptation to human environments. This indicates that the different domestication events for brown rats probably occurred on populations with different levels of pre-adaptation to humans. Genetic analyses of the global commensal populations identify immune system genes as having been positively selected for in populations dispersed from their native range (Zeng et al., 2018). The conditions under which initial captive populations were kept prior to selective breeding will probably have exacerbated this, as rat-catcher accounts describe cages with capacities around 1000 and the individuals being “…piled up with rats, solid…” (Mayhew, 1851: 19). The stock from which the domestic brown rat (in particularly within Europe) derives must thus have undergone 1) significant selective pressure and adaptations to commensal life histories, followed by 2) some likely adaptations to captivity, including to high population density and to rapidly changing environments (Hulme-Beaman et al., 2016). The “wild” state of the immediate ancestors of domesticate rats, then, probably reflected a certain amount of preadaptation to human environments and maybe even human proximity. This has been a particular issue with assessment of domestication experiments and the domestication syndrome, a suite of effects of domestication that are highly debated (Lord et al., 2020; Zeder, 2020). For example, the Russian farm fox experiment, where fur farm foxes were selectively bred for docility towards humans, has been held up as a model for domestication processes, but the animals had previously been kept in non-natural environments (closed and tightly caged), presumably leading to a certain amount of prior adaptations to such conditions (Lord et al., 2020; but see Zeder, 2020). The extent to which domestication syndrome traits emerge or increase in prevalence in commensal or captive (but not domesticated) populations is very unclear, and lacks robust data (Lord et al., 2020; Zeder, 2020), but it is clear that some level of predomestication adaptations or shifts in allelic frequencies occurs in such populations (as seen with brown rats; Zeng et al., 2018).

Early studies have directly compared the physiology of wild commensal rats and domestic rats and found a number of changes. In domestic brown rats, one of the most obvious changes with domestication is the emergence of a wide array of coat colors—white, black, agouti, brown hooded, black hooded, and yellow (see Hedrich, 2019 for full list of strains and colors). Again one can note the early rat-catcher descriptions of different color morphs occurring, albeit rarely, in populations around London (Mayhew, 1851).

A number of organ size changes are also recorded with the adrenal glands, preputial glands, liver, heart, and brain all showing reductions in size (Richter, 1959; Kruska, 1988; Modlinska and Pisula, 2020). The changes in the brain regions in particular reflect reduction in size of brain regions that control motor function (Kruska, 1988). Changes are also present in the reproductive organs, with testes size in young males being larger in domestic rats, whereas testes size in commensal wild rats increases to eventually be larger in old males (Richter, 1959); this may indicate a shift in domestic animals toward sperm competition and rapid reproduction in younger males—a proposed result of domestication occurring in animals domesticated under conditions of higher population density (Hulme-Beaman et al., 2018). This also follows with observations that domestic rats are more gregarious than their wild counterparts (Figure 2C) and mate more readily (Galef et al., 2008). Domestic brown rats are noted as having numerous social and reproductive behavior traits not observed in wild strain colonies, including a heightened level of polyandry, group mating, and mate swapping during copulation (McClintock and Anisko, 1982; Schweinfurth, 2020). Behavior primarily differs between domestic and wild strain populations toward the introduction of unfamiliar rats (individuals that might be considered interlopers in a wild population) (Barnett and Stoddart, 1969; Boreman and Price, 1972). Laboratory strains of domestic brown rats will eventually accept interlopers (Barnett, 1960), whereas laboratory strains of wild brown rats will aggressively attack interlopers, regularly resulting in serious injury (Galef, 1970; Boreman and Price, 1972). These behavioral differences probably reflect the core of the domestication process, although domestication represents a huge range of traits and a continuum rather than an end point, behavioral changes, and the fixation of these in a population is central to the process.

## Conclusions

Examining the pathways rats followed to domestication presents an interesting set of questions that are valuable to consider when trying to understand the concept of domestication and the overall process for other species. Firstly, in brown rats domestication is far more complex than it initially may seem, with multiple possible domestication events that appear to have elements from different pathways. The first, in Japan, appears to have followed a “directed pathway,” but we cannot be sure of the status of their predecessors (i.e. wild, commensal or captive populations). The second and third, in Europe and North America respectively, stem from commensally translocated populations and may have even experienced some selective pressures similar to animals in the “prey pathway”—in which populations are managed prior to directed breeding selections—but under conditions of strict captivity and with subsets of individuals taken for different purposes and under the more clear “directed pathway.” Each of these domestication processes is set against the clear backdrop of this species being highly commensal, and therefore might overall be considered part of the “commensal pathway” to domestication—a pathway which, after all, ordinarily implies eventual direct human intervention at later stages. Of particular relevance here is the fact that the modern domestic rat appears to be exclusively derived from populations that had already undergone commensal adaptations to high population density prior to domestication.

Although the laboratory rat might be the most commonly cited example of domestic brown rats; the rat did not jump straight from its commensal relationship to a laboratory domesticate via strict selective breeding, but rather via a number of intermediate stages for different purposes. The brown rat therefore demonstrates how different requirements of human societies may lead to domestication of the same species under different pathways and different selective processes. Overall, the surviving domestic rat populations of today appear to stem from a commensal/directed pathway; in contrast, had the 1800s rat-baiting colonies survived today the selective pressures involved might be considered close to those we would hypothesize to be associated with the prey pathway. Furthermore, it is not clear whether descendants of the domesticated Japanese Edo period rats still survive today, but they currently represent the earliest evidence for domestication of this species and a discrete domestication center in relation to later events in Europe and North America. The humble domestic brown rat therefore represents all the complexity of human influence on animals both across large geographic distances (different domestication events in different parts of the world) and through time (early domestication in Japan compared with later domestication in Europe and North American) and for different purposes (fancy rats, stock for blood sports and specific experimental breeds). In stark contrast to the enormous amount of knowledge we have of its biology, this review demonstrates we have limited knowledge of many aspects of its origins, with less certainty than is often assumed.

## About the Authors

**Ardern Hulme-Beaman**is a Leverhulme Early Career Fellow based in the Department of Archaeology, Classics and Egyptology at the University of Liverpool. He gained a BA in Archaeology and a MSc in Paleoecology from Durham University. Ardern received a joint PhD from the University of Aberdeen and the Muséum national d’histoire naturelle, Paris, where he studied the human-mediated dispersal of rats and the evolution of commensalism. He previously worked as a postdoctoral research associate on a large project investigating the origins of dog domestication based across the Universities of Aberdeen, Liverpool and Oxford. Ardern primary research interests focus on the evolution and ecology of animals in association with humans. He has expertise in statistical shape analyses and virtual 3D reconstruction, which he applies to study the evolution of feeding and reproductive behavior in human-associated species.

**David Orton** is senior lecturer in zooarcheology in the Department of Archaeology at York, and a member of the BioArCh research group. He has a BA and PhD in archeology from the University of Cambridge and an MSc in zooarcheology from the University of York (a program that he now directs). Before returning to York, he held postdoctoral positions at SUNY Binghamton, Cambridge, and University College London. David works on animal remains from multiple periods and regions across (broadly) Europe, but is particularly interested in early farming in the Neolithic of the Balkans and Anatolia on the one hand, and on animal evidence for urbanism and trade in medieval northern Europe on the other. The latter has involved working extensively on fishing and fish trade and most recently on the European history of black and brown rats.

**Thomas Cucchi** received its PhD from the Natural History Museum of Paris in 2005. He is a zooarcheologist whose research focuses on the origins, spread, and evolution of anthropogenic species over the last 15,000 yr. Using an integrated approach associating zooarcheological studies with the latest development in morphometric and molecular studies, he has published on animal domestication, commensalism, and dispersal in South Western Asia, China, Island South East Asia, and Europe. He is currently working on new methodological approaches of early process of animal domestication in archeology, using functional plasticity in bone morphology and epigenetic markers.
